# PSMB2 plays an oncogenic role in glioma and correlates to the immune microenvironment

**DOI:** 10.1038/s41598-024-56493-5

**Published:** 2024-03-11

**Authors:** Wei He, Zhe Zhang, ZiLong Tan, XinXian Liu, ZeKun Wang, Bo Xiong, XiaoLi Shen, XinGen Zhu

**Affiliations:** 1 Department of Neurosurgery, The Second affiliated hospital, Jiangxi Medical College, Nanchang University, Nanchang, Jiangxi China; 2 Institute of Neuroscience, Nanchang University, Nanchang, Jiangxi China; 3 Department of Neurosurgery, The People’s Hospital of Gao an, Yichun, China

**Keywords:** Glioma, PSMB2, Proliferation, Invasion, Cancer, Biomarkers, Medical research

## Abstract

There has been an upward trend in the incidence of glioma, with high recurrence and high mortality. The beta subunits of the 20S proteasome are encoded by the proteasome beta (PSMB) genes and may affect the proteasome’s function in glioma, assembly and inhibitor binding. This study attempted to reveal the function of the proliferation and invasion of glioma cells, which is affected by proteasome 20S subunit beta 2 (PSMB2). We subjected the data downloaded from the TCGA database to ROC, survival, and enrichment analyses. After establishing the stable PSMB2 knockdown glioma cell line. We detect the changes in the proliferation, invasion and migration of glioma cells by plate colony formation assay, transwell assay, wound healing assay and flow cytometry and PSMB2 expression was verified by quantitative PCR and Western blotting to identify the mRNA and protein levels. PSMB2 expression was higher in glioma tissues, and its expression positively correlated with poor prognosis and high tumor grade and after PSMB2 knockdown, the proliferation, invasion and migration of glioma cells were weakened.

## Introduction

The most common primary intracranial malignant tumor is glioma^1^, and approximately 80% of all malignant central nervous system (CNS) tumors are gliomas; they have a poor prognosis and are highly recurrent. Even though malignant gliomas are treated with surgery, radiotherapy, and chemotherapy by oral drug administration, the prognosis of glioma remains dismal^2^. Therefore, new biological therapeutic targets for glioma patients are important and urgently needed.

PSMB2 (proteasome 20S subunit β2) is a member of the β subunit composing the 20S core structure of the proteasome, which is the catalytic core of the 26S proteasome and the essential enzyme that can degrade ubiquitin-binding proteins^3,4^. The 26S proteasome is an important subapparatus structure in the cell and is composed of core particles with a sedimentation coefficient of 20S and regulatory particles with a sedimentation coefficient of 19S^5^. It plays a corresponding role in protein misfolding and clearing abnormal proteins from aging or oxidative damage^6^. The proteasome also affects cell signal transduction, the cell cycle and proliferation^7^. The function of the proteasome affects patient survival and glioma cell proliferation and invasion, but the mechanism of its action remains unclear^8^. The 20S proteasome consists of four stacked rings each with seven subunits formed by 14 distinct gene products into a four-layered structure^9^. Seven subunits of α and β core particles are encoded by PSMA1-7^10^ and PSMB1-7^11^. Some studies suggest that PSMB2 mainly affects the deactivation and degeneration of various proteins in cancers^12^. Previous studies have indicated that PSMB2 expression in para-cancer tissues is lower than that in tumor tissues, which may be the reason that PSMB2 induces the malignant degree of tumors and various subtle and complex pathways^13,14^. In our analysis, we found that PSMB2 was positively correlated with the malignancy degree of glioma, and it may be highly correlated with the immunity of glioma, indicating its main mechanism. In multiple biological processes of glioma, PSMB2 induced the occurrence and development of glioma. PSMB2 may be engaged in the pathogenesis of glioma, and PSMB2 may play a potential role as a clinical immunotherapeutic target that was not shown in a previous study. Therefore, we analyzed several databases to reveal its role in tumors, which will help us to find a potential immunotherapeutic target, suggesting that PSMB2 is a therapeutic target to alter the progression of glioma.

PSMB2 was suggested as a special prognostic factor for glioma. TCGA database help us to estimate the glioma patient’s expression difference, prognostic value, and biology of PSMB2. Next, to comprehend the contribution of PSMB2 in the glioma pathologic process, we used Gene Ontology (GO) to obtain the cell function. Kyoto Encyclopedia of Genes and Genomes (KEGG) to determine its potential pathway and gene set enrichment (GSEA) to analyze the prognosis of glioma. Next, we detected PSMB2 expression in glioma. The PSMB2 RNA and protein expression in glioma patients was tested by quantitative PCR and Western blotting. Then, we established a clinical model to predict overall survival (OS) at different time points in glioma patients. In conclusion, PSMB2 is expected to be a new focus for glioma immunotherapy.

## Materials and methods

### Datasets

Data for the study were collected from the TCGA database^15^ (https://www.cancer.gov/ccg/). We downloaded the data of glioma patients (n = 706) from TCGA (http://portal.gdc.cancer.gov) to analyze the RNA sequencing and patient information, and we used a transfer tool that was provided by GDC to capture the statistics^16^. In addition, we obtained data from the CGGA database (http://www.cgga.org.cn/) to download PSMB2 expression and clinical data of glioma patients, and analyze the relationship between PSMB2 and patient prognosis. The clinical group of glioma patients was seperated into two subgroups depending on the expression of PSMB2.

### PSMB2 expression between glioma tissue and normal brain tissue

UCSC Xena (http://xena.ucsc.edu) was used to download gene expression data and GTEx^17^. Variance analysis (ANOVA) was used to compare PSMB2 expression in the tumor and corresponding tissue groups. The R language package “ggplot2” was utilized to probe PSMB2 between glioma and brain tissues^18^.

### Survival analysis of PSMB2 in glioma

The information of glioma patients with grades II-IV (WHO) in the TCGA was downloaded to assess the prognostic value of the PSMB2 mRNA of patients with glioblastoma (GBM) and low-grade gliomas (LGG). In addition, we divided samples into two groups based on PSMB2 expression. The R language packages “survival” were used to determine PSMB2 depending on TCGA datasets to build OS conducted by Kaplan‒Meier survival analysis and the Cox proportional hazard model^19^.

### Analysis of DEGs in different groups of glioma patients

The R package “limma” was used to contrast the two groups of PSMB2 to identify the DEGs by the method of unpaired Student’s *t* test. The threshold for DEGs was |log2Fold Change|> 1.5 and adjusted P < 0.05.

### Enrichment analysis of PSMB2

Through the R package “clusterProfiler”, we obtained the cell functions and pathways, and functional enrichment analyses included GO analysis (CC, MF, and BP categories) and KEGG pathway analysis^20^.

### GSEA

The linkage between PSMB2 and glioma prognosis in two PSMB2 subgroups was determined by GSEA to detect an a priori identified set of genes with a statistically significant. In the MSigDB collection, we selected Hallmarks as the reference gene set. We used the R tool ssGSEA and the GSVA package to analyze the linkage between PSMB2 levels and immune infiltration^18^. From published signature gene lists, we determined infiltrating cell types by integrating gene expression levels. The Wilcoxon test and Pearson’s correlation tests were used to determine the linkage between immune cell infiltration and PSMB2 mRNA expression.

### Generation and prediction of prognostic models

We used the Cox proportional hazards model to conduct univariate analyses and multivariate analyses. The overall survival (OS) at 1, 2, and 3 years was predicted by the form of a bar chart, such as 1p/19q codeletion, IDH status, gender, age and race variables.

### Statistical analysis

The correlation between clinicopathologic features and PSMB2 expression was analyzed by several tests, including the Kruskal‒Wallis, Wilcox and chi-square tests. Kaplan‒Meier curves help us to plot survival curves and the log-rank test was used to evaluate differences between two subgroups. Cox models were used to perform the univariate analyses and multivariate analyses. In the multivariate Cox regression analyses, we have 706 data points; information on 11 sample variables was missing, and 695 samples were valid. The standard of statistical significance was P < 0.05.

### PSMB2 and immune-related genes or checkpoints

We used several R packages, including “limma”, “immundeconv”, “Reshape2”, “GGPLOT2” and “Pretty heatmap”, to show the relationship between PSMB2 and immune-related genes or checkpoints. We also evaluated PSMB2 and the response to temozolomide or cisplatin, which are commonly used in glioma, according to one of the largest public pharmacogenomics databases [cancer drugs sensitivity genomics (GDSC), https://www.cancerrxgene.org/], “pRRophetic” forecasts the R package data set for the chemotherapy response of each sample. The maximum half inhibitory concentration (IC_50_) of the samples was estimated by Ridge regression, and the average expression of PSMB2 was used to divide the gliomas into a low expression group and a high expression group. The significance of the samples in the two groups was determined by the Wilcox test. According to the existing chemotherapeutic sensitivity data, we used the “TIDE algorithm” to predict the feasible ICB response, and we forecast accuracy to predict the 50% inhibitory concentration (IC_50_) of drugs by the ridge regression method.

### qRT‒PCR

Total RNA was isolated from normal brain tissues (n = 4), low-grade glioma (n = 14) and glioblastoma (n = 19). TRIzol reagent (Takara, Dalian, China) was used in accordance with the manufacturer’s protocols. Reverse transcription was performed with PrimeScriptTM RT Master Mix (Takara, Dalian, China). qRT‒PCR was conducted with SYBR^®^ Premix Ex Taq™ (Takara, Dalian, China). The relative values for each gene were calculated by the comparative 2 − ΔΔCt method with GAPDH as an internal control. The primers were as follows: PSMB2: F: 5′-3′ ATCCTCGACCGATACTACACAC, R: 5′-3′ GAACACTGAAGGTTGGCAGAT; GAPDH: F: CCCATCACCATCTTCCAGGAG, R: 5′-3′ GTTGTCATGGATGACCTTGGC.

### Western blot analysis

The 33 primary glioma samples were taken from patients who underwent surgical treatment but did not receive chemotherapy or radiotherapy at the Second Affiliated Hospital of Nanchang University between January 2016 and December 2022. The normal brain tissues of 4 patients with traumatic brain surgery were used as control group. All samples were rapidly frozen in liquid nitrogen until total RNA was extracted. Total tissue protein was extracted by cold RIPA lysis buffer (KeyGEN Biotech, China), and we used the BCA method (Takara, Dalian, China) to detect the protein concentration. Proteins with different molecular weights (50 μg) were electrophoresed and electrotransferred to PVDF membranes (Merck Millipore, MA, USA). The membrane was blocked with 5% skim milk at room temperature for 1 h and incubated with specific primary antibodies against PSMB2 (1:500, Abcam, USA) and β-actin (1:1500, ProteinTech, China) at 4 °C overnight. Next, the membrane was paired with the corresponding fluorescent secondary antibody (1:15,000; LI-COR Biosciences, Lincoln, NE, USA), incubated at room temperature for 1 h and visualized with an Odyssey infrared imaging system (LI-COR, Lincoln, NE, USA). Finally, we used ImageJ software to semiquantify the results.

### Cell culture and cell transfection

The glioma cell lines (U87 and U251) were obtained from Cell Bank of the Chinese Academy of Sciences. They were cultured in Dulbecco's modified Eagle’s medium (DMEM, HyClone, United States), supplemented with 10% fetal bovine serum (FBS, Gibco, Grand Island, NY, USA), incubated at 37 ℃ with 5% CO_2_ humidity. Two different small interfering RNAs (siRNAs) against PSMB2 were synthesized by RiboBio (Guangzhou, China) and later transfected into U87 and U251 cells using Lipofectamine 3000 reagent (Invitrogen, CA, USA) according to the manufacturer's instructions. Subsequently, the cells were grouped based on the SiRNA (NC, SiRNA1 against PSMB2 was S1, SiRNA2 against PSMB2 was S2).

### Colony formation assays

Approximately 2000 cells were cultured uniformly in dishes and replaced with 10% FBS medium every 3 days. After two weeks, cells were gently washed two to three times, fixed in PBS, and stained with 4% paraformaldehyde and 0.1% crystal violet. Finally, the cell mass was counted under a microscope.

### Transwell assay

siRNA and were transfected into glioma cells. Then, the bottom surface of chamber was coated with matrigel gel. Medium with 10% FBS was added to the lower chamber and 1 × 10^5^ cell suspension was added to the upper chamber in each treatment group. After incubation for 24 h, the cells in the lower chambers were counted under a light microscope (× 400). A total of 10 transmembrane cells were counted in randomly selected visual fields.

### Wound healing experiment

Glioma cell lines were placed in 6-well plates, and when the cells had grown to approximately 90%, the wound was quickly and steadily aspirated with a sputum suction head. Cell migration ability was observed using a microscope at different time points 0 and 24 and calculated using ImageJ software.

### Flow cytometry

Cells in logarithmic growth phase were seeded in 6-well plates, washed twice with precooled PBS, added with 100 μL loading buffer, then added with 5 μL Annexin V-FITC and PI solution, gently blew and mixed, and incubated at room temperature in the dark for 15 min. The reaction was terminated by adding 400 μL buffer and mixing. The cell suspension was transferred to a 5 mL flow tube in the dark and detected by flow cytometry within 1 h.

### Cell proliferation assay

Cell Proliferation assays were performed on cells using a cell titer 96 (MTS) aqueous reagent (Promega, Madison, WI, USA). 2 × 10^3^ cells were seeded in a 96-well plate, 12 h before the experiment. Then the cells were divided into groups of NC, siRNA1-PSMB2(S1) and siRNA2-PSMB2(S2) and incubated for 0, 24, 48, 72 or 96 h. MTS solution added to each well and incubated for 30 min at 37 ℃. Absorption of triplicate samples was measured at 490 nm using an absorbance reader (Bio-Rad Laboratories, Inc., Hercules, CA, USA).

### Ethical approval

The study was approved by the Ethics Committee of Nanchang University in accordance with the Helsinki Declaration. Written informed consent was obtained from each patient and control subject. All the participants were volunteers.

## Results

### PSMB2, which is related to glioma prognosis, is overexpressed in glioma cells

**Figure 1:**
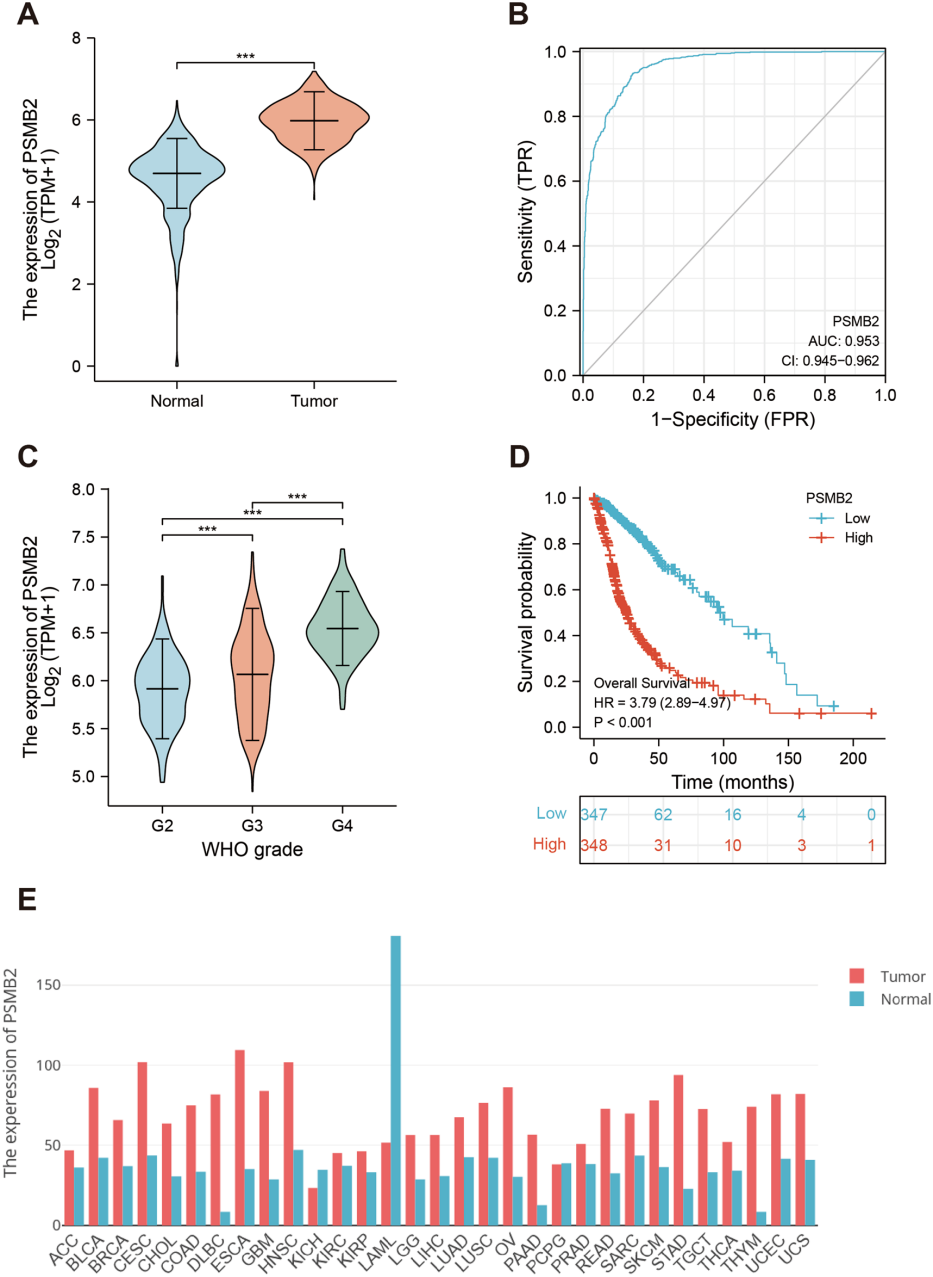
PSMB2 mRNA in glioma and other types of human cancers, survival curves of OS from TCGA data. (**A**) Expression levels of PSMB2 in glioma and normal tissue. (**B**) Receiver operating characteristic analysis (ROC) of PSMB2 in glioma. (**C**) The association of PSMB2 expression and clinical Grade in glioma from TCGA data. (**D**) PSMB2 expression levels in different tumor types from TCGA data. (**E**) Survival curves of OS from TCGA data (n = 706).

PSMB2 expression in glioma cells was significantly higher than that in normal brain cells (Fig. 1A). Further ROC curve analysis showed that AUC value in TCGA dataset was 0.953 (95% CI 0.948–0.968; Fig. 1B); The AUC value in the CGGA dataset was 0.643 (95% CI 0.601–0.686; Supplementary Fig. 1A). In addition, in TCGA database and CGGA database, the expression of PSMB2 was different in different grades of glioma, and its expression increased with increasing malignant degree of the tumor (Fig. 1C, Supplementary Fig. 1B). We found that PSMB2 was overexpressed in multiple malignant tumors in the TCGA database; PSMB2 expression was significantly different between low-grade gliomas and glioblastomas (Fig. 1E).

All samples were separated into two subgroups: a high PSMB2 expression group and a low PSMB2 expression group. In Table 1, we show the specific clinicopathological features of glioma patients. Through Kaplan–Meier survival curve analysis, we found that the higher the expression of PSMB2 in TCGA database and CGGA database, the worse the prognosis of patients. (Fig. 1D, Supplementary Fig. 1C). PSMB2 was considered an independent prognostic factor for OS in glioma through multivariate analysis (HR = 2.807, 95% CI 1.484–5.310, P = 0.002). WHO grade (HR = 1.920, 95% CI 1.253–2.945, P = 0.003, G3 vs. G2) (HR = 4.587, 95% CI 2.707–7.772, P = 0.003, G4 vs. G2), IDH status (HR = 0.296, 95% CI 0.198–0.442, P < 0.001), and age (HR = 1.528, 95% CI 1.122–2.081, P = 0.007) were also considered independent prognostic factors (Supplementary Table 1). Similarly, CGGA database univariate analysis results show that PSMB2 is an independent prognostic factor (Supplementary Table 2).

**Table 1:** Demographic and clinical characteristics of patients with low PSMB2 expression and high expression of glioma in TCGA.

Characteristic	Low expression of PSMB2	High expression of PSMB2	p
n	348	348	
WHO grade, n (%)			< 0.001
G2	166 (26.1%)	58 (9.1%)	
G3	130 (20.5%)	113 (17.8%)	
G4	13 (2%)	155 (24.4%)	
IDH status, n (%)			< 0.001
WT	48 (7%)	198 (28.9%)	
Mut	298 (43.4%)	142 (20.7%)	
1p/19q codeletion, n (%)			< 0.001
Codel	168 (24.4%)	3 (0.4%)	
Non-codel	179 (26%)	339 (49.2%)	
Gender, n (%)			0.818
Female	151 (21.7%)	147 (21.1%)	
Male	197 (28.3%)	201 (28.9%)	
Age, n (%)			< 0.001
≤ 60	298 (42.8%)	255 (36.6%)	
> 60	50 (7.2%)	93 (13.4%)	
OS event, n (%)			< 0.001
Alive	277 (39.8%)	147 (21.1%)	
Dead	71 (10.2%)	201 (28.9%)	
Age, median (IQR)	42 (33, 53)	51 (36, 62)	< 0.001

### Prognostic potential of PSMB2 in glioma cases

**Figure 2:**
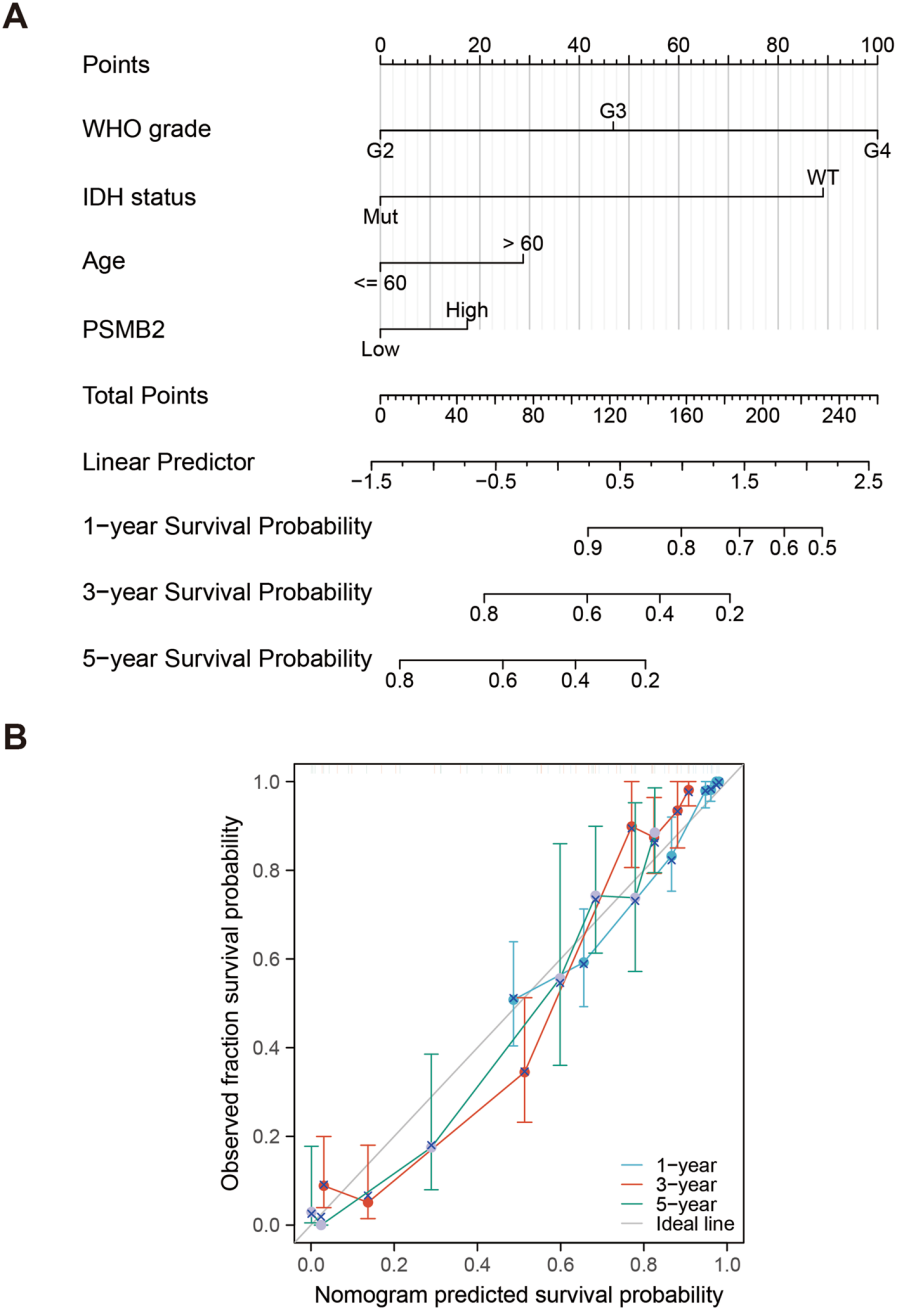
Prognostic model generation and prediction of PSMB2. (**A**) Nomogram integrating PSMB2 and other prognostic factors in glioma from TCGA data. (**B**) nomogram calibration curve.

Clinicopathological parameters and PSMB2 mRNA expression were integrated to construct an OS model depending on the clinicopathological data from TCGA. By incorporating PSMB2 expression and several factors, including WHO, IDH status, 1p/19q codeletion, sex and age, into an OS histogram (Fig. 2A), we found that PSMB2 overexpression was associated with worse tumor prognosis. We calculated the C-index of OS (Fig. 2B) to identify the accuracy of the nomogram. This result indicated that the nomogram built based on PSMB2 expression better predicts survival. Therefore, PSMB2 expression may be a better predictor of glioma patient 1/3/5-year prognosis.

### Functional enrichment analyses by GSEA and GO

The “limma” package was applied to probe high PSMB2 expression groups and low PSMB2 expression groups to reveal the potential mechanism by which PSMB2 accelerates tumor evolvement. A total of 1047 DEGs were identified (Supplementary Fig. 1D–F). The top GO BP, MF, and CC groups were revealed by GO and KEGG analyses, and the results showed that the DEGs of PSMB2 were relevant to the immunoglobulin complex and many other enrichment analysis results (Supplementary Fig. 2A). Furthermore, key pathways associated with PSMB2 were identified by GSEA, and 21 datasets met the requirement (Supplementary Table 3). The most significantly enriched pathways were angiogenesis, epithelial mesenchymal transition, the G2M checkpoint, the interferon gamma response, the interferon alpha response, the IL6/JAK/STAT3 signaling pathway, and the inflammatory response (Supplementary Fig. 2B–G).

### PSMB2 was correlated with infiltration in glioma

KEGG and GSEA analyses revealed the linkage between PSMB2 and the immune microenvironment. We used the ssGSEA calculation to reveal the linkage between PSMB2 and the immune cell infiltration level (Supplementary Table 4) and revealed the subtypes of immune molecules and cell infiltration in tumors. The linkage between PSMB2 and the immune cell infiltration level is display in Supplementary Fig. 3A. PSMB2 was significantly correlated with Th2 cell infiltration levels (R = 0.594, P < 0.001) (Supplementary Fig. 3B), macrophages (R = 0.466, P < 0.001) (Supplementary Fig. 3C), eosinophils (R = 0.384, P < 0.001) (Supplementary Fig. 3D), neutrophils (R = 0.349, P < 0.001) (Supplementary Fig. 3E), aDCs (R = 0.278, P < 0.001) (Supplementary Fig. 3F), iDCs (R = 0.234, P < 0.001) (Supplementary Fig. 3G), T cells (R = 0.234, P < 0.001) (Supplementary Fig. 3H), and T helper cells (R = 0.166, P < 0.001) Supplementary Fig. 3I). ssGSEA also showed that PSMB2 was negatively correlated with the infiltration levels of pDCs (R = − 0.487, P < 0.001) (Supplementary Fig. 3J), NK CD56bright cells (R = − 0.378, P < 0.001) (Supplementary Fig. 3K), TFH cells (R = − 0.239, P < 0.001) (Supplementary Fig. 3L), Tcm cells (R = − 0.239, P < 0.001) (Supplementary Fig. 3M), TReg cells (R = − 0.233, P < 0.001) (Supplementary Fig. 3N), and CD8 T cells (R = − 0.230, P < 0.001) (Supplementary Fig. 3O).

### Relationship between PSMB2 and immune checkpoints and the response to ICB and chemotherapy

**Figure 3:**
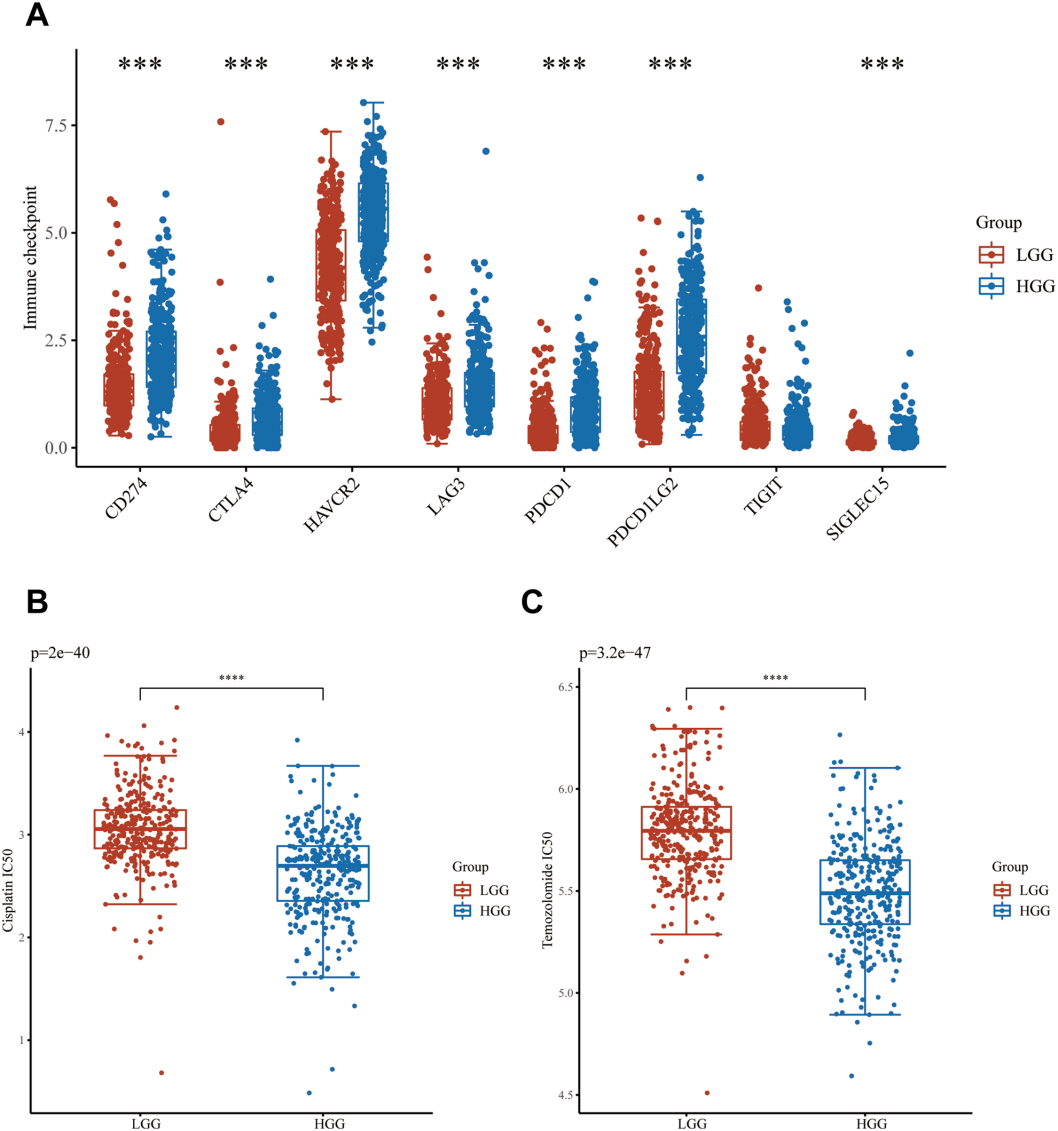
The expression of PSMB2 is related to immune checkpoint, immune checkpoint blocking (ICB) and chemotherapy sensitivity. (**A**) The high PSMB2 expression group and low PSMB2 expression group in glioma patients, the immune checkpoint expression was different between the two groups. (**B**,**C**) Correlation between PSMB2 and chemotherapy drug IC_50_.

To further elucidate the role of PSMB2 in glioma, we also evaluated immune checkpoint differences between PSMB2 in two subgroups. Various checkpoints shown in Fig. 3A were significantly different between the two groups, but TIGHT was not significantly different (Fig. 3A). We also evaluated PSMB2 and the response to temozolomide or cisplatin which are commonly used in glioma, and the lower the expression of PSMB2 was, the higher the IC_50_ values of temozolomide and cisplatin (Fig. 3B,C).

### The expression of PSMB2 in patients with glioma increased with glioma grade

**Figure 4:**
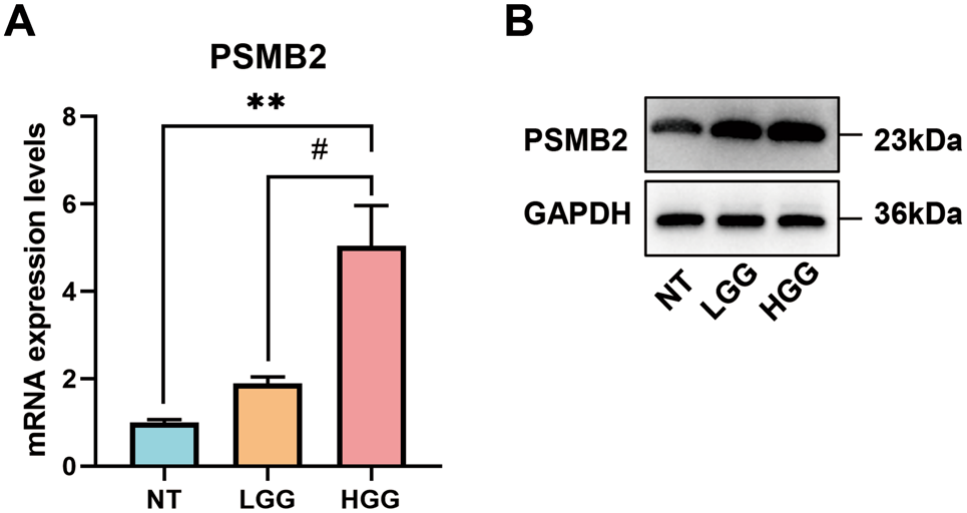
(**A**) PSMB2 mRNA expression in normal tissue (n = 4), LGG tissues (n = 14) and HGG tissues (n = 19). (**B**) PSMB2 protein expression in normal tissue (n = 4), LGG tissues (n = 14) and HGG tissues (n = 19).

We detected the PSMB2 expression level in glioma tissues, 33 glioma tissue specimens (including 14 WHO grade II specimens and 19 WHO grade III-IV specimens) and 4 normal tissue specimens were collected. They were seperated into 3 groups, PSMB2 mRNA expression in gliomas was measured by qRT‒PCR, and PSMB2 mRNA expression in high-grade gliomas was significantly higher than that in low-grade gliomas and normal tissues. PSMB2 protein expression in gliomas was detected by WB, and PSMB2 protein expression in high-grade gliomas was significantly higher than that in low-grade gliomas and normal tissues (Fig. 4A,B).

### PSMB2 silencing inhibits migration, invasion, cell cycle and promotes apoptosis of glioma cells

**Figure 5:**
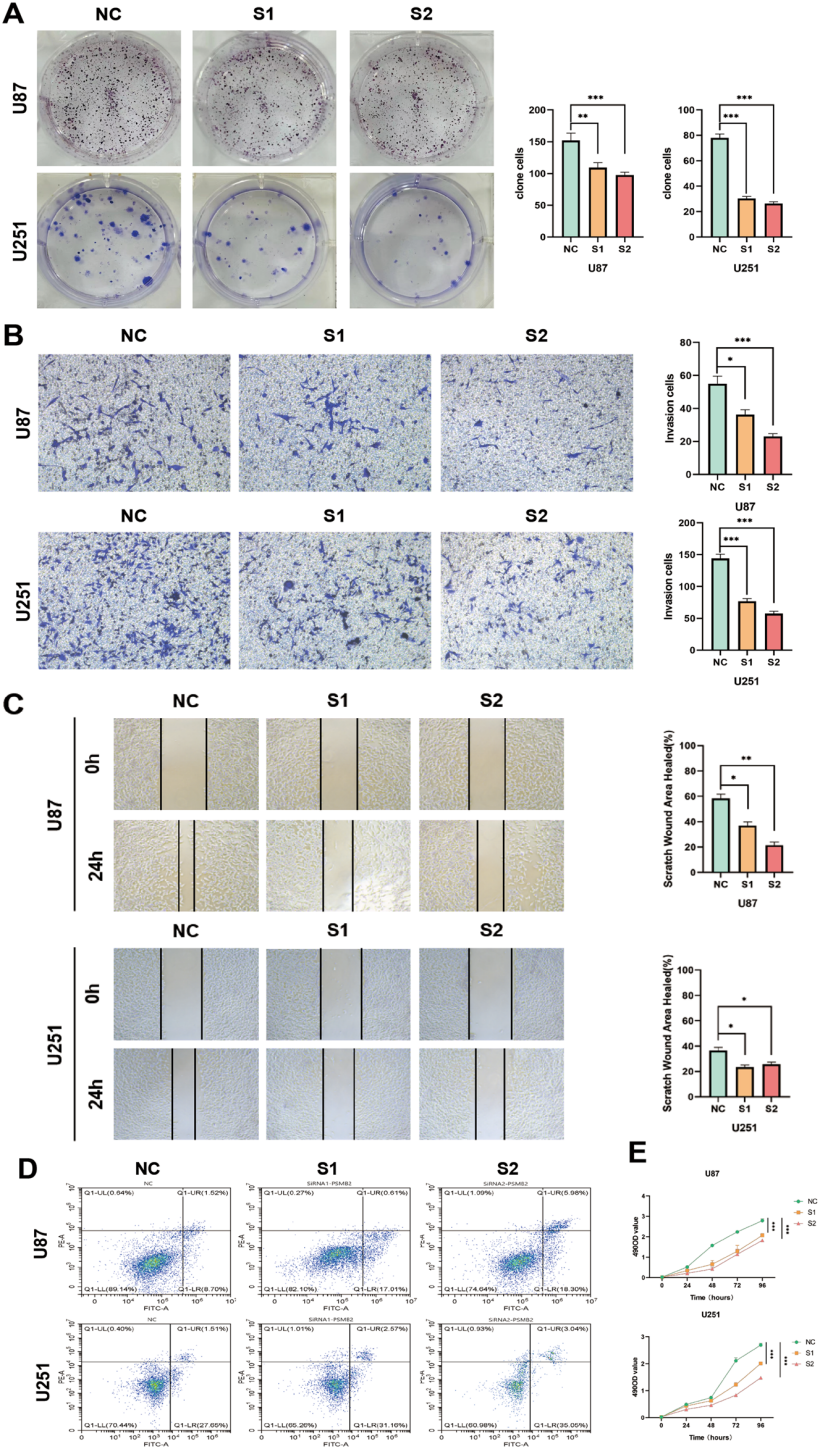
Migration, invasion, cell cycle and apoptosis of glioma cells (S1 = SiRNA1-PSMB2, S2 = SiRNA2-PSMB2). (**A**) Colony formation assay test the proliferation ability of U87 and U251 after knocking down PSMB2. (**B**) Transwell assay test the invasion and migration of U87 and U251 cells after knocking down PSMB2. (**C**) Wound healing assay was used to detect the migration of U87 and U251 in vitro after PSMB2 knockdown. (**D**) Flow cytometry was used to detect the changes of apoptosis of U87 and U251 cells after PSMB2 knockdown. (**E**) The number of proliferation cells significantly decreased in the cells incubated with siPSMB2.

In the Colony formation assays, proliferation ability of U87 and U251 after knocking down PSMB2 were dramatic decline (Fig. 5A). Transwell assay and wound healing assay, we found that the migration and invasion of glioma cells were distinctly inhibited by intervention of siPSMB2 (Fig. 5B,C). And in the flow cytometry, the number of apoptotic cells significantly increased in the cells incubated with siPSMB2 (Fig. 5D). The number of proliferation cells significantly decreased in the cells incubated with siPSMB2 (Fig. 5E).

## Discussion

The clinical treatment of glioma is still a major problem^21^. Conventional surgical treatment has a poor effect, and tumors easily reoccur after surgery. In clinical practice, surgery followed by radiotherapy and chemotherapy is the most commonly used treatment, but the clinical use of various types of chemotherapy drugs for the treatment of glioma is still limited with high mortality and poor prognosis of glioma^22^. Therefore, a stable and effective biomarker is urgently needed in the clinic. In this study, we found that PSMB2 can be used as a good prognostic indicator for clinical prognosis. By analyzing glioma patients’ RNA sequencing data and the clinical outcome data which was downloaded from the TCGA database, we found that PSMB2 was overexpressed in glioma, and PSMB2 expression in glioma increased with increasing tumor malignancy. Moreover, we found that the higher PSMB2 expression was, the shorter the OS of glioma patients. Then, we showed that PSMB2 is highly expressed not only in glioma but also in various tumors through TCGA dataset analysis. We further confirmed the independent prognostic value of PSMB2 by multivariate analysis. The majority of studies have shown that various tumors, including glioma, consider PSMB2 expression an important biomarker for poor prognoses. As PSMB2 is a robust prognostic factor, in this study, PSMB2 expression and clinical information were used to generate a figure to predict glioma patient OS at 1, 2, and 3 years, which is helpful for screening real-threat patients and determining optimal treatment plans for real-threat glioma patients.

PSMB2 is a member of the beta subunit of the proteasome 20S core structure^23^. The 20S proteasome is the catalytic core of the 26S proteasome and the essential enzyme that can degrade ubiquitin-binding proteins^4^. The 26S proteasome is an important intracellular substructure that is composed of a core particle with a sedimentation coefficient of 20S and a regulatory particle with a sedimentation coefficient of 19S^5^. It plays a corresponding role in protein misfolding and clearing abnormal proteins caused by aging or oxidative damage. The proteasome also affects cell signal transduction, the cell cycle, proliferation, etc.^24^. Related studies have also confirmed that PSMB2 affected the cancer cells proliferation and invasion^12^. To further understand the role of PSMB2 in glioma patients, we performed KEGG and GO analyses. PSMB2 is involved in epithelial mesenchymal transition, the G2/M checkpoint, the interferon response, and the IL6/JAK/STAT3 signaling pathway and participates in a series of immune-related processes. The results showed that PSMB2 expression was positively correlated with Th2 cells. Th2 cell infiltration is associated with immunosuppression and affects the prognosis of various tumors. In this study, we found that PSMB2 was overexpressed in glioma patients along with significantly increased infiltration of Th2 cells; consequently, PSMB2 may participate in mediating immune escape in glioma cells. Furthermore, ssGSEA also showed that PSMB2 may regulate immune checkpoint expression, which may promote the activity of chemotherapeutic drugs against tumor cells by enhancing PSMB2 expression and decreasing the therapeutic effects. In our study, we found that high PSMB2 expression may induce the upregulation of CD274, CTLA4, HAVCR2, LAG3, PDCD1, PDCD1LG2 and SIGLEC15, which are molecules on immune cells that facilitate tumor immune escape. The present study also revealed that PSMB2 had adverse effects on the IC_50_ values of temozolomide and cisplatin. It was suggested that high expression of PSMB2 in glioma patients may lead to a better response to temozolomide and cisplatin. Thus, PSMB2 may be a better biomarker of the response to ICB and chemotherapy.

Previous studies have shown that PSMB2 affects tumor invasion, proliferation, the cell cycle and sensitivity to chemotherapy drugs by regulating the tumor microenvironment^25^. The main mechanism may be that PSMB2 participates in the ubiquitin‒proteasome system and plays an important role in the control of the immune response, oxidative stress and maintenance of cellular protein regulation^26^. The ubiquitin‒proteasome system consists of ubiquitin (Ub), ubiquitin activating enzymes (E1), ubiquitin ligases (E2), ubiquitin protein ligases (E3), the 26S proteasome, and deubiquitinating enzymes (DUBs)^27^. Protein degradation by the ubiquitin‒proteasome is a cascade process in which ubiquitinated proteins are finally degraded by the 26S proteasome^28^. In glioma cells, tumor suppressor genes are ubiquitin-labeled and degraded by the 26S proteasome^29^. The 20S proteasome, as its catalytic core, plays an important role in the degradation process of glioma tumor suppressor genes^30^. The lack of the 20S proteasome subunit makes glioma cells easy for the immune system to clear. Therefore, we speculate that the overexpression of PSMB2 can accelerate the degradation of tumor suppressor genes, stabilize the internal environment, promote the proliferation of glioma cells, increase migration and invasion, and inhibit apoptosis. Although this study obtained a deeper understanding of the linkage between PSMB2 and glioma, the role of PSMB2 in glioma progression has not yet been reported.

## Conclusion

PSMB2 mRNA is overexpressed in glioma, and high PSMB2 mRNA is associated with prognosis and OS. PSMB2 has great potential for the prognosis of glioma and may be a new target for glioma immunotherapy.

### Supplementary Information

Supplementary Information.

## Data Availability

The TCGA glioma data used to support the analysis in this study were downloaded from TCGA (http://portal.gdc.cancer.gov); CGGA glioma data were obtained from CGGA (http://www.cgga.org.cn/). Contact the corresponding author via email for requests.
