# Supplementary material for: PSMB2 plays an oncogenic role in glioma and correlates to the immune microenvironment
Source: Sci Rep. 2024 Mar 11;14:5861. doi: 10.1038/s41598-024-56493-5 (PMC10928079; doi:10.1038/s41598-024-56493-5)
Supplement: Supplementary file 1 — Supplementary Information. [file 41598_2024_56493_MOESM1_ESM.pdf]

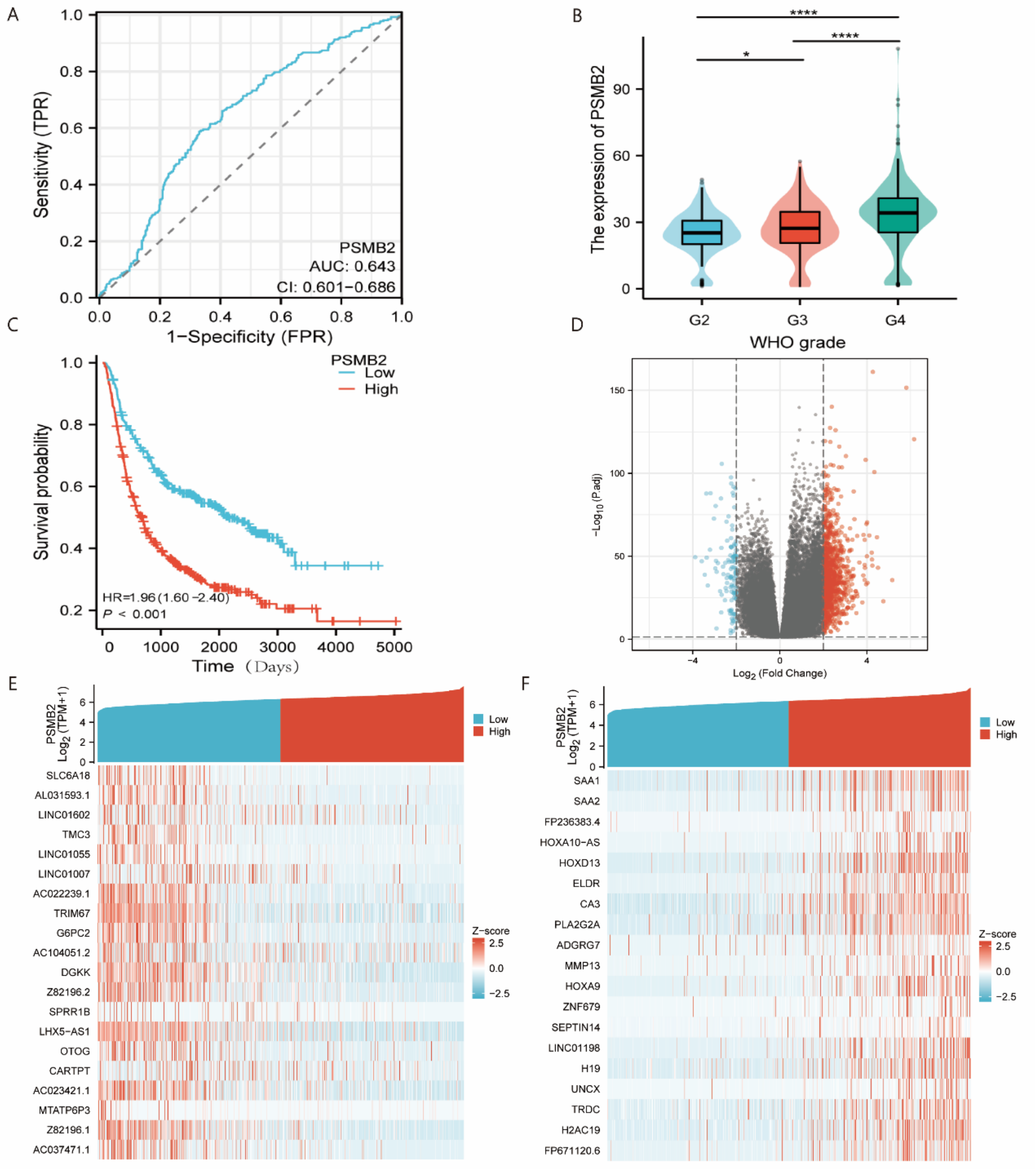

Supplementary Figure 1

(A)Receiver operating characteristic analysis (ROC) of PSMB2 in glioma from CGGA database.

(B)The association of PSMB2 expression and clinical Grade in glioma from CGGA database.

(C)Survival curves of OS from CGGA database.

(D)Volcano maps of differentially expressed gene.

(E-F) Heat maps of differentially expressed genes.

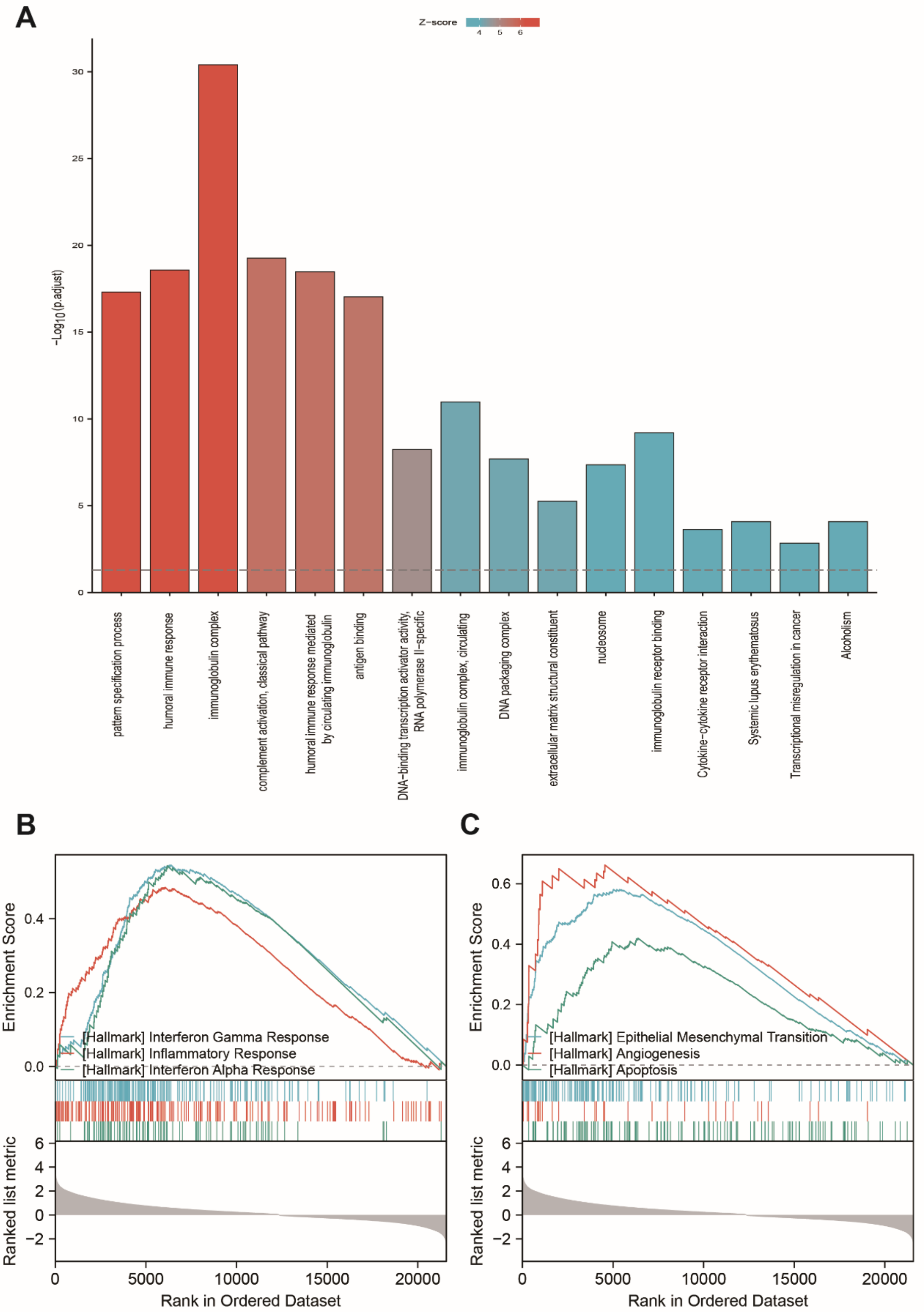

A
Z-score
4
5
6
-Log10(p.adjust)
pattern specification process
humoral immune response
immunoglobulin complex
complement activation, classical pathway
humoral immune response mediated by circulating immunoglobulin
antigen binding
DNA-binding transcription activator activity, RNA polymerase II-specific
immunoglobulin complex, circulating
DNA packaging complex
extracellular matrix structural constituent
nucleosome
immunoglobulin receptor binding
Cytokine-cytokine receptor interaction
Systemic lupus erythematosus
Transcriptional misregulation in cancer
Alcoholism
B
Enrichment Score
[Hallmark] Interferon Gamma Response
[Hallmark] Inflammatory Response
[Hallmark] Interferon Alpha Response
Ranked list metric
Rank in Ordered Dataset
C
Enrichment Score
[Hallmark] Epithelial Mesenchymal Transition
[Hallmark] Angiogenesis
[Hallmark] Apoptosis
Ranked list metric
Rank in Ordered Dataset

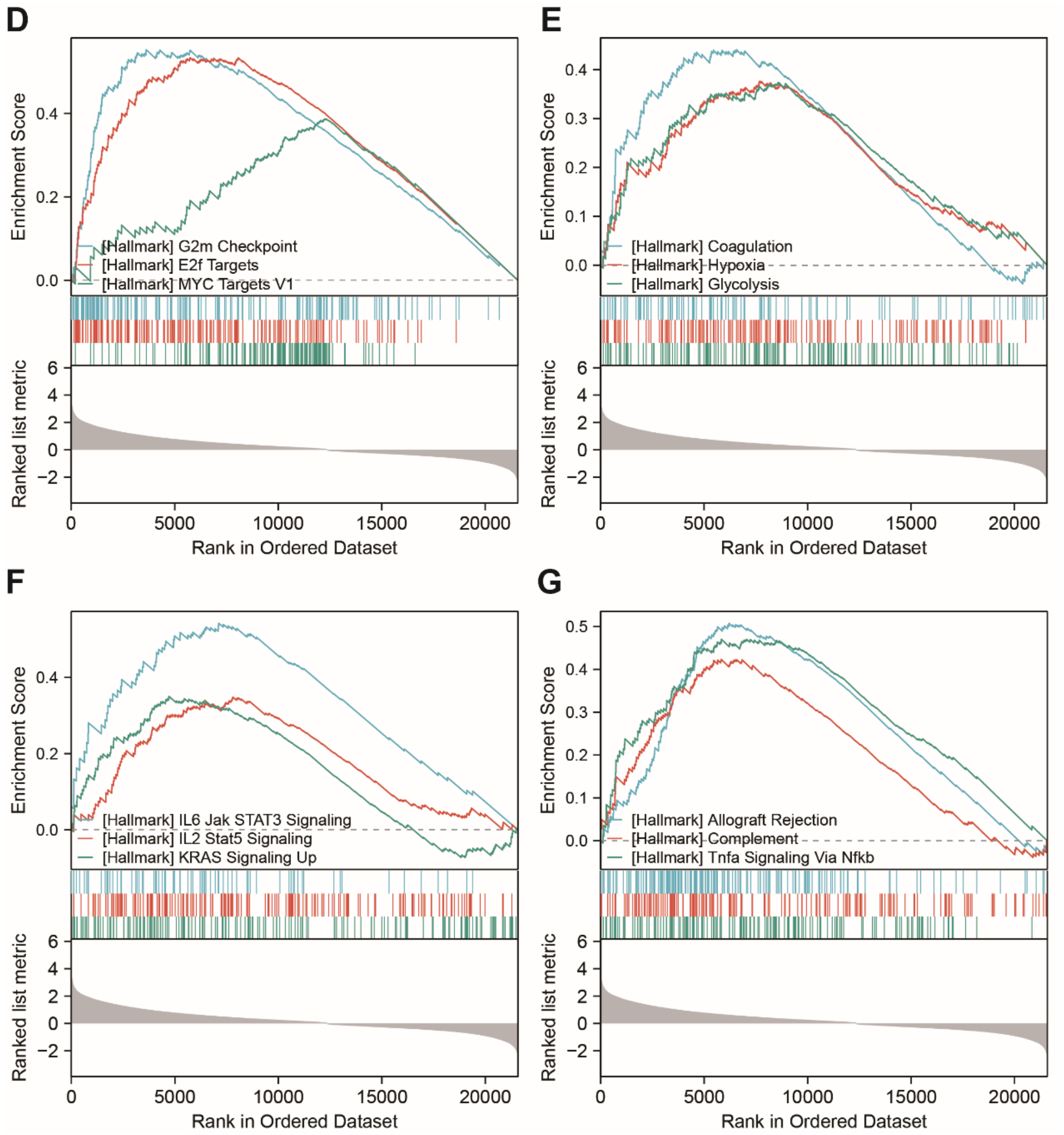

Supplementary Figure 2

Functional enrichment of PSMB2 in glioma.

(A) GO and KEGG enrichment analysis of differentially expressed gene.

(B-G) The most significant enrichment pathways by GSEA.

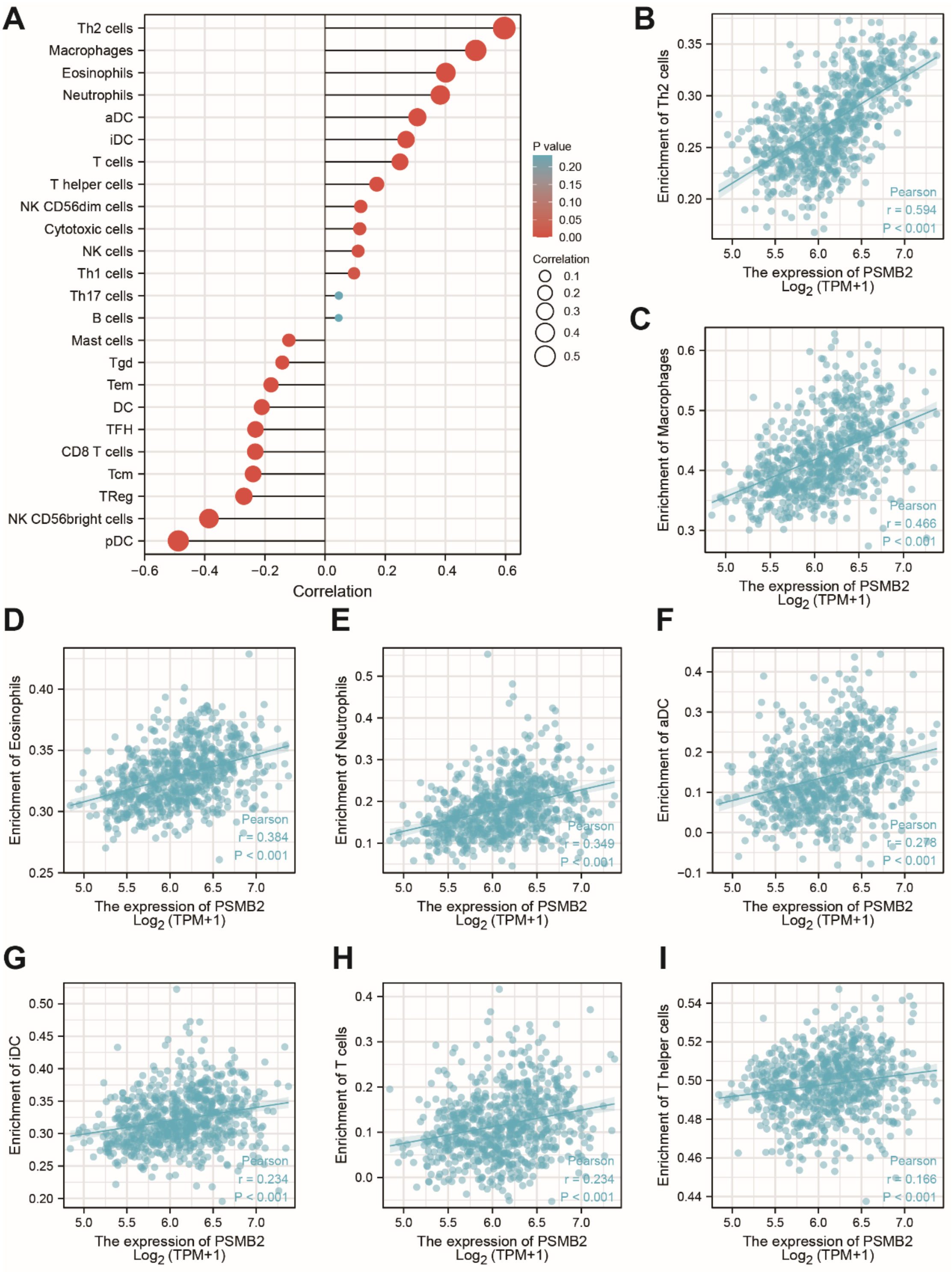
A
Th2 cells
Macrophages
Eosinophils
Neutrophils
aDC
iDC
T cells
T helper cells
NK CD56dim cells
Cytotoxic cells
NK cells
Th1 cells
Th17 cells
B cells
Mast cells
Tgd
Tem
DC
TFH
CD8 T cells
Tcm
TReg
NK CD56bright cells
pDC
−0.6 −0.4 −0.2 0.0 0.2 0.4 0.6
Correlation
P value
0.20
0.15
0.10
0.05
0.00
Correlation
0.1
0.2
0.3
0.4
0.5
B
Enrichment of Th2 cells
Pearson
r = 0.594
P < 0.001
The expression of PSMB2
Log2 (TPM+1)
C
Enrichment of Macrophages
Pearson
r = 0.466
P < 0.001
The expression of PSMB2
Log2 (TPM+1)
D
Enrichment of Eosinophils
Pearson
r = 0.384
P < 0.001
The expression of PSMB2
Log2 (TPM+1)
E
Enrichment of Neutrophils
Pearson
r = 0.349
P < 0.001
The expression of PSMB2
Log2 (TPM+1)
F
Enrichment of aDC
Pearson
r = 0.278
P < 0.001
The expression of PSMB2
Log2 (TPM+1)
G
Enrichment of iDC
Pearson
r = 0.234
P < 0.001
The expression of PSMB2
Log2 (TPM+1)
H
Enrichment of T cells
Pearson
r = 0.234
P < 0.001
The expression of PSMB2
Log2 (TPM+1)
I
Enrichment of T helper cells
Pearson
r = 0.166
P < 0.001
The expression of PSMB2
Log2 (TPM+1)

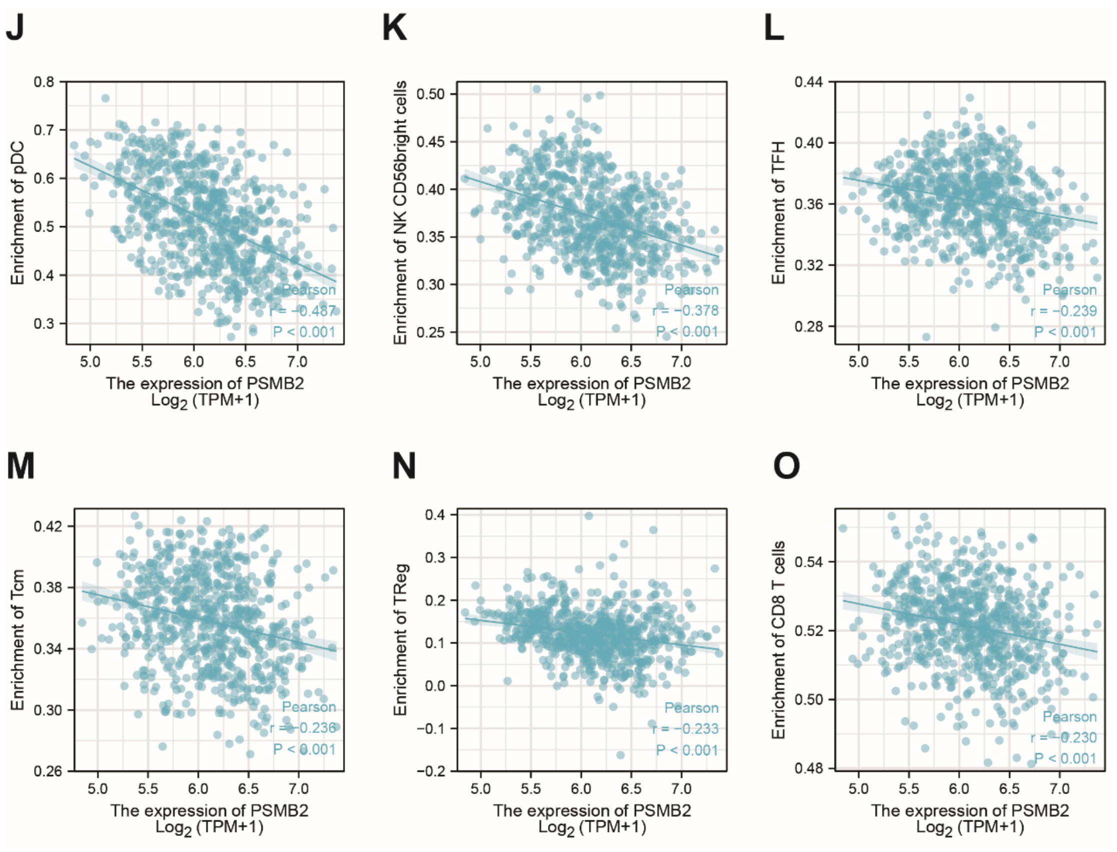

Supplementary Figure 3 ssGSEA analyses of PSMB2 and the correlation of PSMB2 expression with immune infiltration level in glioma.

(A) The correlation between the infiltration of immune cells and the expression of PSMB2.

(B-I) PSMB2 expression significantly positively correlates with infiltrating levels of Th2 cells, Macrophages, Eosinophils, Neutrophils, aDC, iDC, T cells, T helper cells.

(J-O) PSMB2 expression significantly negatively correlates with infiltrating levels of pDC , NK CD56bright cells, TFH, Tcm, TReg, CD8 T cells.

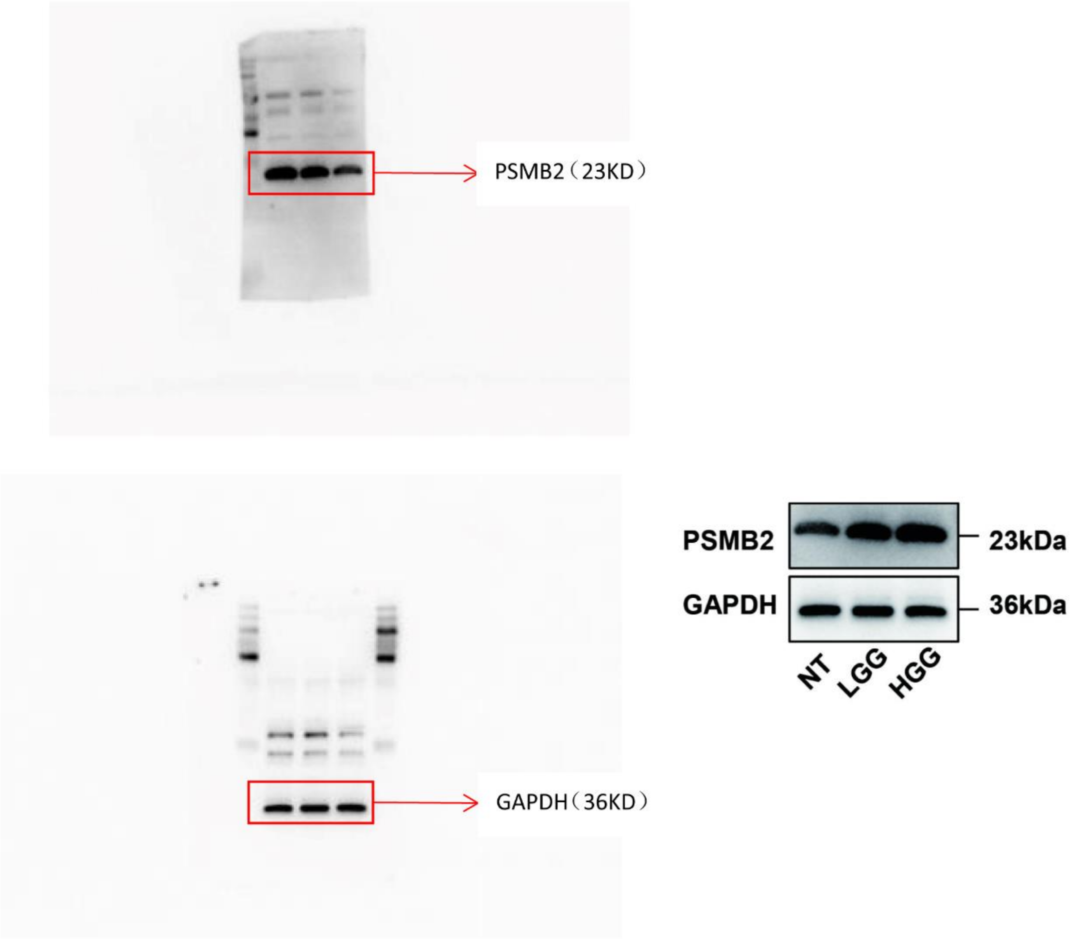

Supplementary Figure 4 WB full film

Supplementary Table 1

The univariate and multivariate analysis of overall survival according to PSMB2 expression in TCGA dataset (n=695)

Characteristics	Total(N)	Univariate analysis		Multivariate analysis	
		Hazard ratio (95% CI)	P value	Hazard ratio (95% CI)	P value
WHO grade	634				
G2	223	Reference			
G3	243	2.999 (2.007-4.480)	**<0.001**	1.920 (1.253-2.945)	**0.003**
G4	168	18.615 (12.460-27.812)	**<0.001**	4.587 (2.707-7.772)	**<0.001**
IDH status	685				

Characteristics	Total(N)	Univariate analysis		Multivariate analysis	
		Hazard ratio (95% CI)	P value	Hazard ratio (95% CI)	P value
WT	246	Reference			
Mut	439	0.117 (0.090-0.152)	**<0.001**	0.296 (0.198-0.442)	**<0.001**
Gender	695				
Female	297	Reference			
Male	398	1.262 (0.988-1.610)	0.062	1.210 (0.921-1.589)	0.171
Age	695				
<=60	552	Reference			
>60	143	4.668 (3.598-6.056)	**<0.001**	1.528 (1.122-2.081)	**0.007**
1p/19q codeletion	688				
codel	170	Reference			
non-codel	518	4.428 (2.885-6.799)	**<0.001**	1.315 (0.762-2.268)	0.326
Race	682				
Asian&Black or African American	46	Reference			
White	636	0.821 (0.502-1.344)	0.433		
PSMB2	695				
Low	347	Reference			
High	348	5.950 (4.493-7.879)	**<0.001**	2.807 (1.484-5.310)	**0.002**

Supplementary Table 2

The univariate analysis of overall survival according to PSMB2 expression in CGGA dataset (n=656)

**Characteristics**	**Total(N)**	**Univariate analysis**	
		**Hazard ratio (95% CI)**	**P value**
**Age**	656		

<= 60	588	Reference	
> 60	68	0.671 (0.583 - 0.771)	**< 0.001**
**Gender**	656		
Female	283	Reference	
Male	373	0.969 (0.876 - 1.071)	0.537
**IDH status**	608		
Mut	333	Reference	
WT	275	0.733 (0.605 - 0.888)	**0.001**
**1p/19q codeletion**	590		
Codel	137	Reference	
Non-codel	453	0.442 (0.351 - 0.557)	**< 0.001**
**WHO grade**	656		
G2	172	Reference	
G3	247	0.383 (0.314 - 0.467)	**< 0.001**
G4	237	0.980 (0.843 - 1.138)	**0.787**
**PSMB2**	656		
Low	328	Reference	
High	328	2.032 (1.660 - 2.488)	**< 0.001**

Supplementary Table 3

Hallmark pathways enriched in high-risk groups and low-risk groups by using GSEA.

Description	NES	pvalue	p.adjust
HALLMARK_EPITHELIAL_MESENCHYMAL_TRANSITION	2.24384284	0.00101523	0.00451427
HALLMARK_E2F_TARGETS	2.06432984	0.00101626	0.00451427
HALLMARK_G2M_CHECKPOINT	2.13640963	0.00101626	0.00451427
HALLMARK_INTERFERON_GAMMA_RESPONSE	2.11858047	0.00101626	0.00451427
HALLMARK_ALLOGRAFT_REJECTION	1.96608531	0.00101729	0.00451427

HALLMARK_COMPLEMENT	1.635308<br>36	0.001017<br>29	0.004514<br>27
HALLMARK_INFLAMMATORY_RESPONSE	1.878430<br>62	0.001017<br>29	0.004514<br>27
HALLMARK_TNFA_SIGNALING_VIA_NFKB	1.815474<br>29	0.001017<br>29	0.004514<br>27
HALLMARK_APOPTOSIS	1.593936<br>17	0.001030<br>93	0.004514<br>27
HALLMARK_COAGULATION	1.642056<br>19	0.001050<br>42	0.004514<br>27
HALLMARK_INTERFERON_ALPHA_RESPONSE	1.991396<br>37	0.001062<br>7	0.004514<br>27
HALLMARK_IL6_JAK_STAT3_SIGNALING	1.942932<br>31	0.001083<br>42	0.004514<br>27
HALLMARK_ANGIOGENESIS	2.065880<br>71	0.001209<br>19	0.004650<br>73
HALLMARK_MYC_TARGETS_V1	1.483351<br>92	0.003064<br>35	0.010944<br>11
HALLMARK_HYPOXIA	1.454644<br>96	0.005086<br>47	0.016954<br>9
HALLMARK_GLYCOLYSIS	1.435635<br>75	0.008146<br>64	0.025458<br>25
HALLMARK_KRAS_SIGNALING_UP	1.360327<br>33	0.019308<br>94	0.056791<br>01
HALLMARK_IL2_STAT5_SIGNALING	1.353965<br>92	0.022357<br>72	0.062104<br>79
HALLMARK_KRAS_SIGNALING_DN	1.716386<br>08	0.043478<br>26	0.114416<br>48
HALLMARK_MITOTIC_SPINDLE	1.301287<br>7	0.046795<br>52	0.116988<br>81
HALLMARK_PANCREAS_BETA_CELLS	1.471011<br>35	0.053254<br>44	0.126796<br>28

Supplementary Table 4

Relationship between PSMB2 mRNA expression and immune cell infiltration level

PSMB2	Cells	Correlation (Pearson)	P-value (Pearson)	Correlation (Spearman)	P-value (Spearman)
	aDC	0.278	<0.001	0.307	<0.001
	CD8 T cells	-0.230	<0.001	-0.232	<0.001
	DC	-0.150	<0.001	-0.211	<0.001
	Eosinophils	0.384	<0.001	0.401	<0.001
	iDC	0.234	<0.001	0.269	<0.001
	Macrophages	0.466	<0.001	0.501	<0.001
	Neutrophils	0.349	<0.001	0.383	<0.001
	NK CD56bright cells	-0.378	<0.001	-0.386	<0.001
	pDC	-0.487	<0.001	-0.488	<0.001
	T cells	0.234	<0.001	0.249	<0.001
	T helper cells	0.166	<0.001	0.172	<0.001
	Tcm	-0.236	<0.001	-0.239	<0.001
	Tem	-0.166	<0.001	-0.180	<0.001
	TFH	-0.239	<0.001	-0.232	<0.001
	Tgd	-0.142	<0.001	-0.142	<0.001
	Th2 cells	0.594	<0.001	0.596	<0.001
	TReg	-0.233	<0.001	-0.270	<0.001
	Mast cells	-0.120	0.002	-0.120	0.001
	Cytotoxic cells	0.102	0.007	0.115	0.002
	NK CD56dim cells	0.123	0.001	0.119	0.002
	NK cells	0.105	0.005	0.110	0.004
	Th1 cells	0.097	0.010	0.096	0.011
	Th17 cells	0.032	0.395	0.046	0.228
	B cells	0.035	0.362	0.045	0.233
